# Molecular interaction of inhibitors with human brain butyrylcholinesterase

**DOI:** 10.17179/excli2021-4418

**Published:** 2021-11-25

**Authors:** Shazi Shakil

**Affiliations:** 1 King Fahd Medical Research Center, King Abdulaziz University, Jeddah, Saudi Arabia; 2 Department of Medical Laboratory Technology, Faculty of Applied Medical Sciences, King Abdulaziz University, Jeddah, Saudi Arabia

**Keywords:** Alzheimer's disease, butyrylcholinesterase, MD simulation, protein ligand docking, screening cascade

## Abstract

Alzheimer's disease is a topic of deep research interest across the global scientific community. Butyrylcholinesterase (BuChE) is an important enzyme, and an interesting anti-Alzheimer's target. Identification or fresh design of promising BuChE-inhibitors is warranted. Virtual screening supported by molecular dynamics simulations has emerged as a key component of present drug-discovery cascades. The research piece aimed at identification of a putative BuChE-inhibitor as a fresh molecular frame that might aid drug design in the context of Alzheimer's disease. The study utilized 'MCULE' to screen a set of 5 million ligands to test their ability to bind to human BuChE. Pharmacokinetic profiling was achieved by the 'SWISS ADME' program. Toxicities were duly assessed. YASARA STRUCTURE version 20.10.4.W.64 was employed to run 133 ns molecular dynamics (MD) simulation for the complex of 'the top screened out inhibitor' and 'the human BuChE enzyme'. The simulation was executed for approx. 4 days (~93 hrs) on an HP ZR30w workstation. YANACONDA, a special language contained in YASARA STRUCTURE was employed to perform complex tasks. Fine resolution figures (notably the RMSD vs time plot) were created. Snapshots were extracted at every 250 ps. The selected ligand, (3-Bromophenyl)[5-(4-chlorophenyl)-5-hydroxy-3-(trifluoromethyl)-4,5-dihydro-1H-pyrazol-1-yl]methanone, exhibited the best overall binding with human BuChE. It interacted with human BuChE through 19 residues. Markedly, 9 of the 19 residues were confirmed to be matching to those of the reference complex (PDB ID 5DYW). Trajectory analysis returned 533 snapshots. The RMSD versus time plot indicated that around 22 ns, equilibrium was achieved and, from then on, the 'BuChE-Top inhibitor' complex remained predominantly stable. From 22 ns and onwards till 133 ns, the backbone RMSD fluctuations were observed to remain limited within a range of 1.2-1.9 Å. The molecule, (3-Bromophenyl)[5-(4-chlorophenyl)-5-hydroxy-3-(trifluoromethyl)-4,5-dihydro-1H-pyrazol-1-yl]methanone, satisfied ADMET requirements. Additionally, the feasibility of the proposed enzyme-inhibitor complex was supported by an adequately extended MD simulation of 133 ns. Hence, the proposed molecule could be a likely lead for designing inhibitor(s) against human BuChE. Scope remains for validatory wet laboratory investigation.

## Introduction

Alzheimer's disease is a topic of deep research interest across the global scientific community. Occurrence of this disorder in the elderly is well-known. It is known to lead to progressive dementia and at times to death. Butyrylcholinesterase (BuChE) is an important enzyme in the context of Alzheimer's disease (Miles et al., 2020[14]). Butyrylcholinesterase is basically an enzyme which is a serine hydrolase (Darvesh et al., 2003[2]). Authors have stated that BuChE might contribute as much or more to the metabolism of acetylcholine than acetylcholinesterase performs in late Alzheimer's disease; and that this has led to the consideration of BuChE inhibition as a symptomatic treatment for the same (Miles et al., 2020[14]). Scientists across the world are trying to discover fresh lead molecules for design of selective inhibitors of the aforementioned neuroenzyme (Kamal et al., 2017[6]; Miles et al., 2020[14]). Previously, we had explored binding interactions of 'Galangin' with butyrylcholinesterase (Shaikh et al., 2014[17]). Similarly other scientists, like Kumar et al. have reported new derivative ligands based on the 2-phenylbenzofuran backbone (Kumar et al., 2018[11]). Likewise, further search needs to be performed concerning more drug like molecules to look into their probable binding to Butyrylcholinesterase so as to find promising 'seed' molecules for drug design. Identification or fresh design of promising butyrylcholinesterase-inhibitors continues to be a hot topic among researchers in the context of Alzheimer's disease (Miles et al., 2020[14]). Virtual screening supported by molecular dynamics simulations has emerged as a key component of present drug-discovery cascades (Rizvi et al., 2013[16]; Shakil, 2020[18]; Shakil et al., 2021[22]). The current requirement for an efficient butyrylcholinesterase-inhibitor is understandable. The research piece aimed at identification of a putative BuChE-inhibitor as a fresh molecular frame that might aid drug design in the context of Alzheimer's disease. In this study, pertinent in silico methods including virtual screening, docking, SWISS ADME profiling, TOX CHECK and a comprehensive 133 ns molecular dynamics simulation were used to identify an efficient butyrylcholinesterase-inhibitor.

## Methods

### Exploring the binding spot

The PDB ID 5DYW was used as the reference complex in this study. It is the complex of human BuChE (protein) and N-((1-benzylpiperidin-3-yl)methyl)-N-(2-methoxyethyl)naphthalene-2-sulfonamide (ligand). Three-dimensional structural features of the binding site in the aforementioned complex were carefully explored by 'CASTp3.0' (Tian et al., 2018[23]). This program is capable of offering comprehensive information about the binding site for an enzyme-inhibitor complex. It employs the 'α-shape method' for ascertaining molecular features (Tian et al., 2018[23]).

### Screening

The study utilized 'MCULE' to screen a large set of ligands (Kiss et al., 2012[7]). The 'InChIKey' of the ligand contained in the said reference complex was fetched to 'Chemspider' which in turn generated its detailed properties by using 'ACD/Labs Percepta Platform - PhysChem Module'. Based on these properties, the input range (i.e. the minimum and maximum values) was constructed and entered in the MCULE screening workflow (Kiss et al., 2012[7]). Five million test molecules were screened for their ability to successfully bind to human BuChE. Concisely, '1 violation of the famous Lipinski's rule (RO5)' was permitted, thereby imparting certain broadness to the early screens. The value for maximum number of rotatable bonds was entered as 9. The values of the minima and maxima for mass and polar surface area were entered as 422.61-482.61 Da and 38-78 Å^2^, respectively. Similarly the minima and maxima for hydrogen bond acceptors were kept as 3 and 8, respectively; while for hydrogen bond donors the assigned range was 0-3. A value of 1000 was assigned against the 'sampler size' while a value of 3 million was entered in 'the maximum number of compounds after sphere exclusion' tab (displayed in the MCULE workflow builder). The 'cut off threshold for similarity' was fixed at 0.85. OBLF (Open Babel Linear Fingerprint) was employed for analysis of molecular-descriptors, while the rest of the parameters were maintained at their default values in the MCULE platform. 

### Protein-ligand docking

The 3 D structure file for butyrylcholinesterase was extracted from PDB ID 5DYW by Discovery Studio Visualizer 4.1. The protein pdb was fetched to the 'MCULE workflow' which subsequently implemented AutoDock Vina (Trott and Olson, 2010[24]). The volume of the docking grid was kept as 60 Å^3^. The three position co-ordinates specified for docking had the values as 16.283906, -24.849469 and -41.551094 for x, y and z, respectively. The values were determined by analyzing the reference pdb file 5DYW. 

### Autodock VINA ranks and pharmacokinetic profiles

The candidate small molecules were rated by VINA scores (Trott and Olson, 2010[24]). Accordingly, putative inhibitors that were ranked higher by VINA were recorded. These small molecules were subjected to SWISS ADME pharmacokinetic analyses (Daina et al., 2017[1]). An array of assessments, namely success or failure to pass through the Lipinski, Ghose, Veber, Egan, PAINS as well as Muegge filter, was made for the candidate molecules. These filters basically check the likelihood of the small molecules to behave as putative drugs (Ghose et al., 1999[4]; Egan et al., 2000[3]; Muegge et al., 2001[15]; Veber et al., 2002[25]; Lipinsky, 2004[12]). Those test molecules that acquired the top VINA ranks and additionally displayed successful passage through at least 4 of the above mentioned filters were recorded. 

### Dock Score cut off, no violation of 'Lipinsky rule of five', toxicity assessment and ease of synthesis

The upper layer of top 50 ligands (out of the complete set of screened out ligands) was considered for further investigation. The test molecules that displayed a 'VINA-docking score' greater than -10.1 were excluded. The remaining set of ligands was subjected to pharmacokinetic profiling by SWISS ADME program (Daina et al., 2017[1]). Importantly, all of these ligand structures were carefully checked for their possible toxicity in the human body. The 'toxicity checker' tool within the MCULE platform was employed for the purpose (Kiss et al., 2012[7]). The factor of 'ease of synthesis' was also considered using a scoring function (Daina et al., 2017[1]). Further, the candidate inhibitors were checked for their ability for crossing Blood Brain Barrier (BBB). Finally, a ligand that displayed overall most acceptable pharmacokinetic properties (and notably exhibited zero 'RO5 violation') was designated as the 'Top putative BuChE inhibitor' in the present study. 

### Binding contacts of the 'Top putative BuChE inhibitor' and 'molecular overlay' analysis 

The residues involved in the binding interaction of the top putative inhibitor with BuChE enzyme were marked using Discovery Studio Visualizer (version 4.1). Moreover, the aforementioned complex was compared against the reference complex having the PDB ID 5DYW with aid of 'molecular overlay' figure constructed by the Discovery Studio Visualizer (version 4.1). 

### YASARA molecular dynamics simulation (133 ns)

YASARA STRUCTURE version 20.10.4.W.64 was employed to run 3 replicas of molecular dynamics (MD) simulation for the complex of 'the top screened out inhibitor' and 'the human BuChE enzyme' (Krieger and Vriend, 2014[10]). The experimental setup comprised of H-bond optimization as well as pKa prediction for the selected pH (i.e. 7.4) (Krieger et al., 2012[8]). This was followed by addition of NaCl ions (0.9 %), cell neutralization, and minimization of energy. The latter in turn ensured geometry correction of the structure. Conformational stress was removed employing “a short steepest descent minimization”. Further, this process sustained through “simulated annealing” that involved a time step of 2 femtoseconds and scaling down of the 'atom velocities' by 0.9 at every 10^th^ step until attaining convergence. After these aforementioned initial procedures, the MD simulation was run for 133 ns employing the AMBER14 (Maier et al., 2015[13]) for the solute, GAFF2 (Wang et al., 2004[26]) as well as AM1BCC (Jakalian et al., 2002[5]) for ligand, and TIP3P for water. The setup used temperature and pressure values as 298 Kelvin and one atmosphere, correspondingly. Relevant algorithms stay defined previously (Krieger and Vriend, 2015[9]). Trajectory was analyzed with the aid of a 'macro' in YASARA, namely 'md_analyze'. YANACONDA, a special language contained in YASARA STRUCTURE was employed to perform complex tasks related to the MD simulation. Fine resolution figures (notably the RMSD vs time plot) were created in the YASARA interface (v.20.10.4.W.64). Simulation snapshots were extracted at every 250 ps, thereby generating 533 snapshots for a comprehensive analysis.

## Results and Discussion

### The binding spot

Molecular level exploration of the reference crystal, 5DYW concentrated on the residues holding the ligand, using a probing radius of 1.4 Å in CASTp3.0, showed that the binding contacts comprised of 18 amino acid residues (Tian et al., 2018[23]). The names of the residues as revealed by Discovery Studio Visualizer were N68, I69, W82, G116, G117, Q119, S198, W231, L286, S287, A328, F329, Y332, F398, W430, M437, H438 and Y440.

### Results of the screening cascade

**Figure 1:**
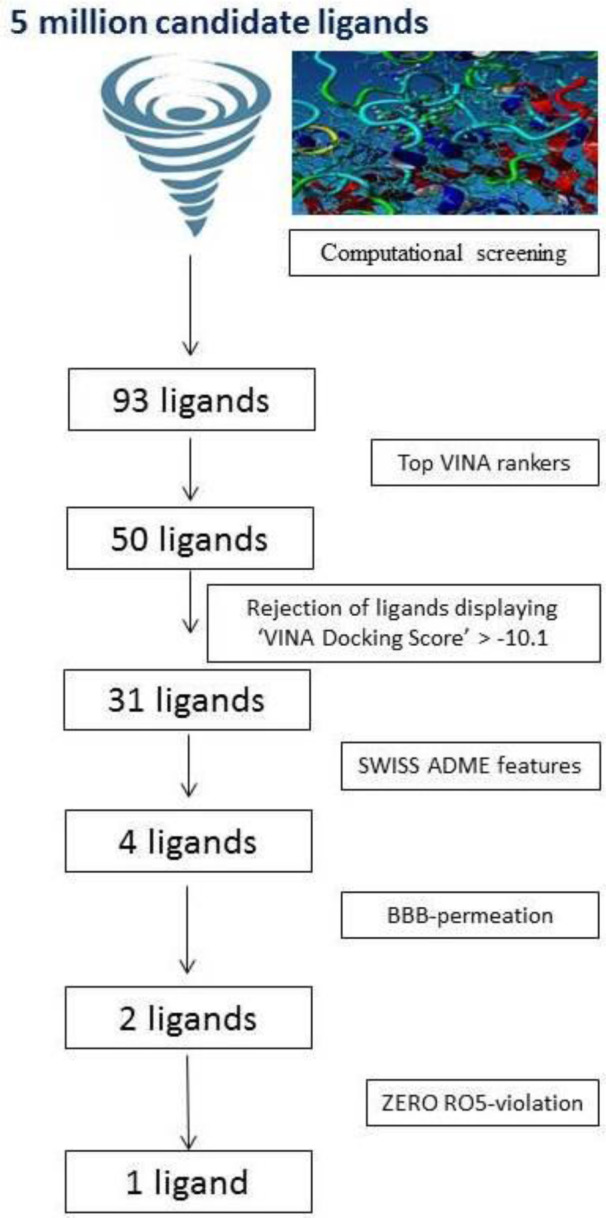
Molecular screening cascade for 5 million candidate ligands targeted against butyrylcholinesterase enzyme

Molecular screening was grounded on the 3-D-structure of BuChE, the target protein. In this research work, a total of five million candidate ligands were tested for their binding with human BuChE. This revealed a set of 93 ligands of interest (Figure 1(Fig. 1)).

### Autodock VINA ranks and results of pharmacokinetic profiling

It is noteworthy that the author has a global rank #1 under the research keywords 'Protein ligand docking' on Google Scholar (https://scholar.google.com/citations?view_op=search_authors&hl=en&mauthors=label:protein_ligand_docking; Accession Date: 07 Oct 2021). Previous successful publications related to molecular interactions fuelled the inspiration for the present work (Rizvi et al., 2013[16]; Shakil et al., 2019[21], 2021[22]; Shakil, 2020[18]). Importantly, the works described the interactions involving important neuroenzymes like acetylcholinesterase and butylcholinesterase (Kamal et al., 2017[6]; Shakil, 2017[19]; Shakil, 2019[20]). Herein, Vina was used for molecular screening (executed within the MCULE drug discovery platform). This program is well-known about its ability for expressively improving the overall accuracy of the docking (Trott and Olsen, 2010[24]).

Not surprisingly, VINA still remains one of the most popular programs for in silico screening of huge ligand sets (Trott and Olson, 2010[24]). Upper 'VINA-docking-scores' are usually indicative of better 'fitting' of the ligands in the site of interest. Accordingly, 50 ligands exhibiting the top layer of 'VINA-docking-scores' were obtained. 

### Further filtration (Dock score cut off, RO5 violation check, toxicity assessment and ease of synthesis)

Further, the test molecules that displayed a 'VINA-docking score' greater than -10.1 were excluded. Hence, only 31 ligands remained for further investigation. This remaining set of ligands was subjected to pharmacokinetic profiling by SWISS ADME program for additional filtration (Daina et al., 2017[1]). Molecular structures which exhibit poor pharmacokinetic characteristics are usually precluded at the very onset of the drug-design route. To ensure flexibility at the beginning of the screening cascade, any of the ligands that happened to pass at least 4 of the 7 filters named in Table 1(Tab. 1), i.e. Lipinski, Ghose, Veber, Egan, Muegge, PAINS and Brenk were accepted for further investigation (Ghose et al., 1999[4]; Egan et al., 2000[3]; Muegge et al., 2001[15]; Veber et al., 2002[25]; Lipinsky, 2004[12]). For similar reason, single RO5-violation was ignored at the beginning. All of these ligand structures were carefully checked for their possible toxicity in the human body. The 'toxicity checker' tool within the MCULE platform was employed (Kiss et al., 2012[7]). Moreover, the factor of 'ease of synthesis' was also considered using a scoring function (Daina et al., 2017[1]). In this manner, 4 ligands (out of 31) were chosen. 'SWISS ADME' profiling of the 4 top scoring putative inhibitors of butyrylcholinesterase is shown in Table 1(Tab. 1).

**Figure 2:**
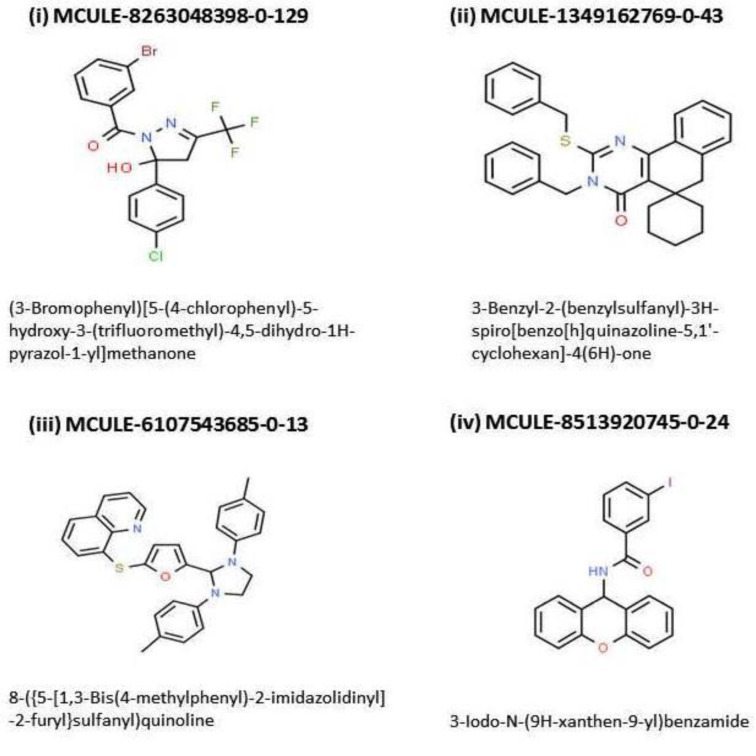
Molecular structures of the 4 top ranking ligands obtained from the screening cascade

The 4 candidate inhibitors were identified as: MCULE-8263048398-0-129, MCULE-1349162769-0-43, MCULE-6107543685-0-13, and MCULE-8513920745-0-24. It is noteworthy that all of these four ligands were able to pass the toxicity checking filter of the MCULE platform. Albeit, 2 of these four ligands namely, MCULE-1349162769-0-43 and MCULE-6107543685-0-13 were found to be unable to permeate the BBB, and hence were rejected; thereby leaving only two ligands (MCULE-8263048398-0-129 and MCULE-8513920745-0-24) for further consideration. Only one of the two remaining ligands i.e. MCULE-8263048398-0-129 displayed absolutely zero RO5 violation. The corresponding IUPAC name for MCULE-8263048398-0-129 was found to be (3-Bromophenyl)[5-(4-chlorophenyl)-5-hydroxy-3-(trifluoromethyl)-4,5-dihydro-1H-pyrazol-1-yl]methanone (Figure 2(Fig. 2), molecule (i). 

It exhibited a high predicted gastrointestinal absorption, a positive pointer for possible oral administration. Moreover, MCULE-8263048398-0-129 was predicted to be BBB permeable, yet again a positive feature for a putative neurodrug. Consequently, MCULE-8263048398-0-129 or (3-Bromophenyl)[5-(4-chlorophenyl)-5-hydroxy-3-(trifluoromethyl)-4,5-dihydro-1H-pyrazol-1-yl]methanone emerged as the 'Top putative BuChE inhibitor' in the present study.

### Results of the 'molecular overlay' analysis

**Figure 3:**
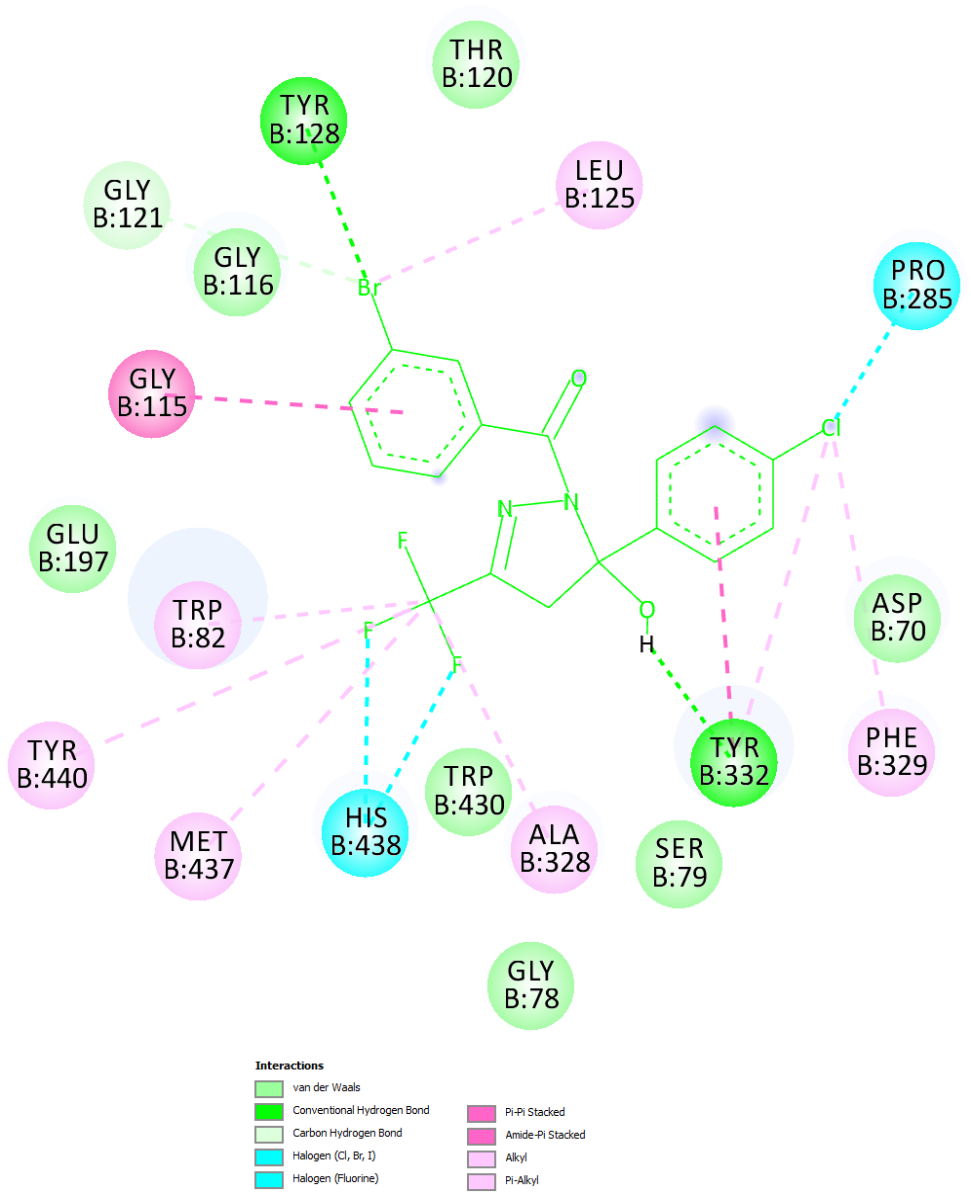
'2-D-Diagram' corresponding to the 'butyrylcholinesterase-top inhibitor'-complex generated by 'Discovery Studio Visualizer' [BIOVIA]

Figure 2(Fig. 2) shows the molecular structures of the 4 top ranking ligands obtained from the screening cascade while the Figure 3(Fig. 3) presents the '2-D-Diagram' corresponding to the 'butyrylcholinesterase-top inhibitor'-complex generated by 'Discovery Studio Visualizer' [BIOVIA] (Figures 2(Fig. 2) and 3(Fig. 3)).

**Figure 4:**
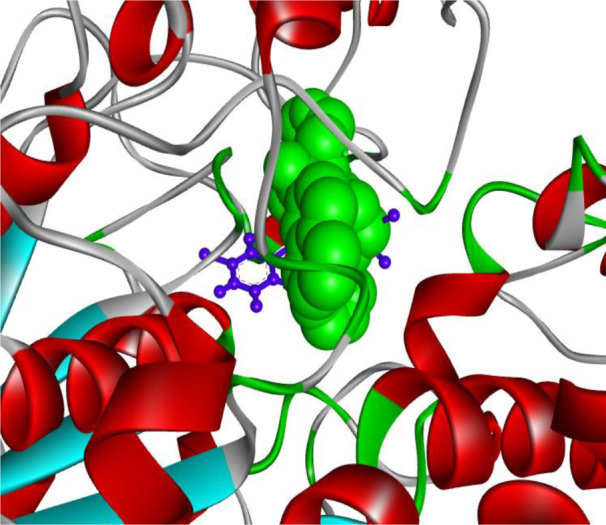
'Molecular overlay' of the 'top inhibitor' bound to butyrylcholinesterase in conjunction with '5 HF' (used as a reference molecule). The 'top inhibitor' and the 'reference inhibitor' are presented in 'ball & stick' (blue) and CPK (green) styles, respectively.

The binding contact residues are labeled. The top putative inhibitor, (3-Bromophenyl)[5-(4-chlorophenyl)-5-hydroxy-3-(trifluoromethyl)-4,5-dihydro-1H-pyrazol-1-yl]methanone interacted with BuChE protein through 19 residues. Significantly, 9 of the 19 residues were confirmed to be matching to those of the reference complex. These matching amino acid residues were found to be W82, G116, A328, F329, Y332, W430, M437, H438 and Y440. Importantly, the binding crevice for the ligand present in the reference complex (PDB ID 5DYW) and that for the 'Top putative BuChE inhibitor' were found to be the same; as also pointed by the 9 common residues mentioned above. Further to this, the aforementioned complex was compared against the reference complex having the PDB ID 5DYW with aid of 'molecular overlay' figure constructed by the Discovery Studio Visualizer (version 4.1). The 'molecular overlay' of the 'top inhibitor' bound to butyrylcholinesterase in conjunction with '5 HF' (used as a reference molecule) is depicted (Figure 4(Fig. 4)).

### Results of YASARA molecular dynamics simulation (133 ns)

**Figure 5:**
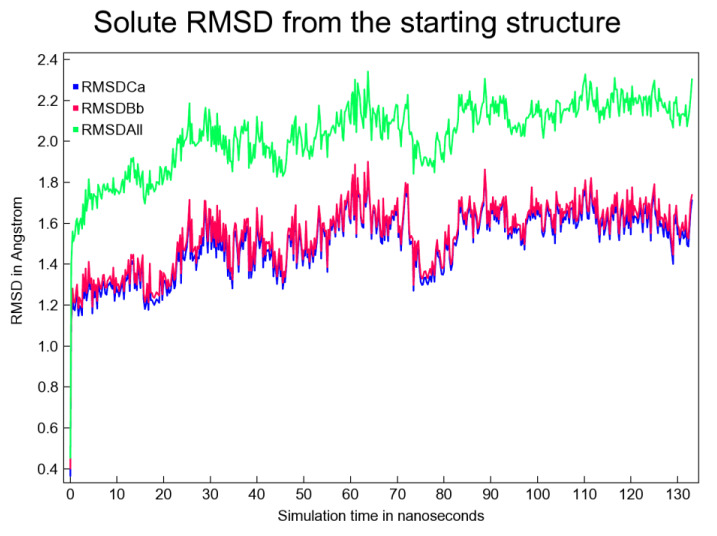
Solute RMSD (measured from the initial structure) plotted as a function of simulation time

The trajectory was analyzed by YASARA STRUCTURE v.20.10.4.W.64 for 133 ns duration employing AMBER14. A total of 533 snapshots were extracted from the MD simulation. The simulation was executed for approx. 4 days (~93 hrs) on an HP ZR30w workstation. The simulation snapshots were extracted at every 250 ps. The simulation was repeated at least 3 times, which further confirmed the findings. Several interesting figures like the 'ray-traced diagram' of the 'BuChE-Top putative inhibitor' complex subjected to simulation; another ray-traced figure for the 'Top putative BuChE inhibitor' and also the plot showing the fluctuations in the total potential energy as simulation proceeded, were also generated. Other important plots like 'Per-residue number of contacts as a function of simulation time' were also obtained. The number of contacts indicated as to how densely a particular residue range was packed and allowed identification of important binding residues (e.g. Phenylalanine). However, as a generalization, residues displaying 0 contacts are quite rare and these are often glycine residues. Evolution of binding contact patterns of BuChE and the top inhibitor with time was also mapped, whereby H-bonds, hydrophobic and ionic interactions were plotted in red, green, and blue colored dots, respectively. The aforementioned plots have not been shown here. Figure 5(Fig. 5) presents the solute RMSD versus time plot.

The RMSD versus time plot shown above indicated that around 22 ns, equilibrium was achieved and, from then on, the 'BuChE-Top inhibitor' complex remained predominantly stable. From 22 ns and onwards till 133 ns, the backbone RMSD fluctuations were observed to remain limited within a range of 1.2-1.9 Å. This slender range was considered tolerable for this plot (Figure 5(Fig. 5)). The root mean square fluctuation (RMSF) plot as well as the 'dynamic cross-correlation matrix' was also prepared (Figures not shown). All these plots taken together indicate the feasibility of the putative complex. 

### Limitation of the study

A limitation of the study is that the identified molecule has not yet been experimentally verified in wet laboratory.

## Conclusions

This research work presents the computational binding interactions of 'human BuChE enzyme' with a fresh putative inhibitor, chosen out of 5 million candidate ligands by an in silico screening cascade. The chosen molecule, (3-Bromophenyl)[5-(4-chlorophenyl)-5-hydroxy-3-(trifluoromethyl)-4,5-dihydro-1H-pyrazol-1-yl]methanone, satisfied ADMET requirements. Additionally, the feasibility of the proposed enzyme-inhibitor complex was supported by an adequately extended MD simulation of 133 ns. 

Therefore, (3-Bromophenyl)[5-(4-chlorophenyl)-5-hydroxy-3-(trifluoromethyl)-4,5-dihydro-1H-pyrazol-1-yl]methanone could be likely lead for designing inhibitor(s) against human BuChE enzyme. Scope remains for detailed wet laboratory investigation to validate the computational findings. 

## Declaration

### Author contributions

All the work described in the present article was performed in its entirety by SS, as the sole author.

### Funding

This research was funded by the Deanship of Scientific Research (DSR) at King Abdulaziz University, Jeddah, under grant no. G: 421-142-1440. 

### Institutional Review Board Statement

Not applicable.

### Informed Consent Statement

Not applicable.

### Acknowledgment

This project was funded by the Deanship of Scientific Research (DSR) at King Abdulaziz University, Jeddah, under grant no. G: 421-142-1440. The author, therefore, acknowledges with thanks DSR for technical and financial support.

### Conflict of interest

The author declares no conflict of interest.

## Figures and Tables

**Table 1:**
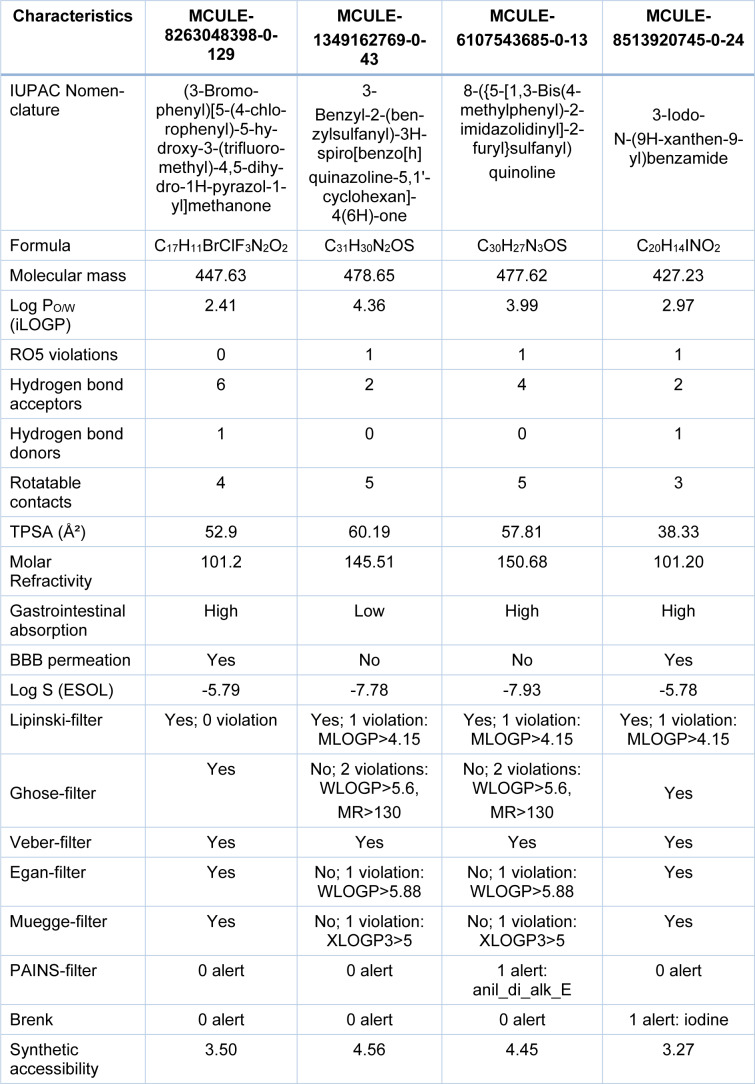
'SWISS ADME' profiling of the 4 top scoring putative inhibitors of butyrylcholinesterase

## References

[1] Daina A, Michielin O, Zoete V (2017). SwissADME: a free web tool to evaluate pharmacokinetics, drug-likeness and medicinal chemistry friendliness of small molecules. Sci Rep.

[2] Darvesh S, Hopkins DA, Geula C (2003). Neurobiology of butyrylcholinesterase. Nat Rev Neurosci.

[3] Egan WJ, Merz KM, Baldwin JJ (2000). Prediction of drug absorption using multivariate statistics. J Med Chem.

[4] Ghose AK, Viswanadhan VN, Wendoloski JJ (1999). A knowledge-based approach in designing combinatorial or medicinal chemistry libraries for drug discovery. 1. A qualitative and quantitative characterization of known drug databases. J Comb Chem.

[5] Jakalian A, Jack DB, Bayly CI (2002). Fast, efficient generation of high-quality atomic charges. AM1-BCC model: II. Parameterization and validation. J Comput Chem.

[6] Kamal MA, Shakil S, Nawaz MS, Yu QS, Tweedie D, Tan Y (2017). Inhibition of butyrylcholinesterase with fluorobenzylcymserine, an experimental alzheimer's drug candidate: validation of enzoinformatics results by classical and innovative enzyme kinetic analyses. CNS Neurol Disord Drug Targets.

[7] Kiss R, Sandor M, Szalai FA (2012). http://Mcule.com: a public web service for drug discovery. J Cheminform.

[8] Krieger E, Dunbrack RL, Hooft RW, Krieger B (2012). Assignment of protonation states in proteins and ligands: combining pKa prediction with hydrogen bonding network optimization. Methods Mol Biol.

[9] Krieger E, Vriend G (2015). New ways to boost molecular dynamics simulations. J Comput Chem.

[10] Krieger E, Vriend G (2014). YASARA View - molecular graphics for all devices - from smartphones to workstations. Bioinformatics.

[11] Kumar A, Pintus F, Di Petrillo A, Medda R, Caria P, Matos MJ (2018). Novel 2-pheynlbenzofuran derivatives as selective butyrylcholinesterase inhibitors for Alzheimer's disease. Sci Rep.

[12] Lipinski CA (2004). Lead- and drug-like compounds: the rule-of-five revolution. Drug Discov Today Technol.

[13] Maier JA, Martinez C, Kasavajhala K, Wickstrom L, Hauser KE, Simmerling C (2015). ff14SB: Improving the accuracy of protein side chain and backbone parameters from ff99SB. J Chem Theory Comput.

[14] Miles JA, Kapure JS, Deora GS, Courageux C, Igert A, Dias J (2020). Rapid discovery of a selective butyrylcholinesterase inhibitor using structure-based virtual screening. Bioorg Med Chem Lett.

[15] Muegge I, Heald SL, Brittelli D (2001). Simple selection criteria for drug-like chemical matter. J Med Chem.

[16] Rizvi SM, Shakil S, Haneef M (2013). A simple click by click protocol to perform docking: AutoDock 4.2 made easy for non-bioinformaticians. EXCLI J.

[17] Shaikh S, Ahmad SS, Ansari MA, Shakil S, Rizvi SM, Shakil S (2014). Prediction of comparative inhibition efficiency for a novel natural ligand, galangin against human brain acetylcholinesterase, butyrylcholinesterase and 5-lipoxygenase: a neuroinformatics study. CNS Neurol Disord Drug Targets.

[18] Shakil S (2020). Molecular interaction of anti-cancer ligands with human brain acetylcholinesterase. J Biomol Struct Dyn.

[19] Shakil S (2017). Molecular interaction of anti-diabetic drugs with acetylcholinesterase and sodium glucose co-transporter 2. J Cell Biochem.

[20] Shakil S (2019). Molecular interaction of investigational ligands with human brain acetylcholinesterase. J Cell Biochem.

[21] Shakil S, Baig MH, Tabrez S, Rizvi SMD, Zaidi SK, Ashraf GM (2019). Molecular and enzoinformatics perspectives of targeting Polo-like kinase 1 in cancer therapy. Semin Cancer Biol.

[22] Shakil S, Rizvi SMD, Greig NH (2021). High throughput virtual screening and molecular dynamics simulation for identifying a putative inhibitor of bacterial CTX-M-15. Antibiotics (Basel).

[23] Tian W, Chen C, Lei X, Zhao J, Liang J (2018). CASTp 3.0: computed atlas of surface topography of proteins. Nucleic Acids Res.

[24] Trott O, Olson AJ (2010). AutoDock Vina: improving the speed and accuracy of docking with a new scoring function, efficient optimization, and multithreading. J Comput Chem.

[25] Veber DF, Johnson SR, Cheng HY, Smith BR, Ward KW, Kopple KD (2002). Molecular properties that influence the oral bioavailability of drug candidates. J Med Chem.

[26] Wang J, Wolf RM, Caldwell JW, Kollman PA, Case DA (2004). Development and testing of a general amber force field. J Comput Chem.

